# Evaluating the real-world safety of cholestyramine for the treatment of hyperlipidemia: disproportionality analysis of FAERS data

**DOI:** 10.3389/fmed.2026.1765949

**Published:** 2026-02-11

**Authors:** Qiang Li, Mengmeng Fan, Anbang Gao, Limin Qin

**Affiliations:** 1 Department of Cardiology, Affiliated Hospital of Shandong Second Medical University, Weifang, Shandong, China; 2 Department of Neonatology, Weifang Maternal and Child Health Hospital, Weifang, Shandong, China; 3 Department of Emergency, Affiliated Hospital of Shandong Second Medical University, Weifang, Shandong, China

**Keywords:** adverse reactions, cholestyramine, disproportionality analysis, FAERS, hypercholesterolemia

## Abstract

**Background:**

Hypercholesterolemia is a significant risk factor for severe cardiovascular diseases. Cholestyramine lowers serum low-density lipoprotein cholesterol (LDL-C) levels and is clinically indicated for the treatment of primary hypercholesterolemia, relieve itching symptoms caused by bile acid accumulation in cholestatic diseases (such as primary biliary cirrhosis), as well as to manage bile acid diarrhea resulting from bile acid metabolic disorders. With its widespread clinical application, it is essential to understand its safety in real-world settings.

**Methods:**

This study evaluated the clinical safety of cholestyramine by analyzing all adverse event reports since 2004 in the FDA Adverse Event Reporting System (FAERS), where cholestyramine was identified as the primary suspected drug. Bayesian Confidence Propagation Neural Network (BCPNN), the Medicines and Healthcare Products Regulatory Agency (MHRA) composite criteria method, Multi-Item Gamma Poisson Shrinker (MGPS), Proportional Reporting Ratio (PRR), and Reporting Odds Ratio (ROR) were used to analyze adverse events associated with cholestyramine.

**Results:**

The study results confirmed known adverse reactions of cholestyramine, such as constipation, abdominal discomfort, bloating, steatorrhea, bleeding tendencies, night blindness, hyperchloremic acidosis, osteoporosis, rashes, and local irritation caused by deficiencies in vitamins K, A, and D, which are also listed in the drug’s package insert. Additionally, adverse reactions not documented in the package insert were identified, including off-label use, administration for unapproved indications, gastroesophageal reflux disease (GERD), irritable bowel syndrome (IBS), fecal abnormalities (color changes, softening, hardening), blood glucose fluctuations, tooth fracture, and exacerbation of concurrent medical conditions. This study also underscores the importance of early detection of adverse reactions associated with cholestyramine.

**Conclusion:**

By providing insights into both known and potential adverse reactionsin real-world settings, the findings offer enhanced safety information to assist clinicians in prescribing cholestyramine for conditions such as hypercholesterolemia, cholestasis-associated pruritus, and bile acid diarrhea.

## 1 Introduction

Coronary heart disease (CHD) is one of the most common and severe cardiovascular disease. Its high incidence and case-fatality rate threaten patients’ lives, markedly degrade quality of life, and impose a heavy burden on the health-care systems (1). Hypercholesterolemia, particularly elevated levels of low-density lipoprotein cholesterol (LDL-C), is an important risk factor for CHD and myocardial infarction (MI) (2). Excess LDL-C deposits in the coronary intima, forming atherosclerotic plaques that narrow or occlude vessels, precipitating myocardial ischaemia, angina, and potentially MI. Consequently, is essential to prevent cardiovascular events, Excess LDL-C deposits in the coronary intima, forming atherosclerotic plaques that narrow or occlude vessels, precipitating myocardial ischemia, angina, and potentially MI (3). Consequently, aggressive lipid management, and in particular maintaining LDL-C within target ranges, is crucial for preventing cardiovascular events, retard atherogenesis, and reduce CHD morbidity and mortality.

Cholestyramine, an anion-exchange resin that sequesters bile acid in the gut, interrupts their enterohepatic circulation.

The drug is indicated for primary hypercholesterolaemia—particularly in statin-intolerant patients—and for cholestatic pruritus (e.g., in primary biliary cholangitis) and bile-acid diarrhoea caused by malabsorption. Although its efficacy is well established, safety concerns persist. By binding intestinal bile acids, cholestyramine can impair fat digestion and cause bloating, constipation, or diarrhoea. It also reduces absorption of fat-soluble vitamins (A, D, E, and K), potentially leading to osteoporosis (vitamin D deficiency) or bleeding disorders (vitamin K deficiency).

The resulting bile-acid depletion drives hepatic conversion of cholesterol to new bile acids, thereby reducing serum LDL-C levels (4). It is clinically used primarily for the treatment of primary hypercholesterolemia, especially in patients who are intolerant to statins (4, 5). It is also used to relieve itching symptoms caused by bile acid accumulation in cholestatic diseases (such as primary biliary cirrhosis) and to manage bile acid diarrhea resulting from bile acid malabsorption (6–8). Although the efficacy of cholestyramine is well-established, safety concerns persist. By binding intestinal bile acids, cholestyramine can impair fat digestion and cause constipation, bloating or diarrhea (9). It also reduces the absorption of fat-soluble vitamins (A, D, E, and K), increasing the risk of deficiencies in these vitamins (10). This can, in turn, potentially leading to osteoporosis (vitamin D deficiency) or coagulation disorders (vitamin K deficiency).

The FDA adverse event reporting system (FAERS) is a global pharmacovigilance database that collects spontaneously reported adverse events (AEs) from healthcare professionals, patients, and pharmaceutical companies to detect post-marketing safety signals. Compared with clinical trials, FAERS offers larger sample sizes, longer observation, and greater population diversity, making it particularly effective in detecting rare or delayed adverse events. Data-mining of FAERS, for example, researchers have successfully identified mental status changes, conversion disorder, and eye movement disorder caused by Regadenoson, highlighting its value in pharmacovigilance (11). As clinical experience accumulates, FAERS mining suggests that the full safety profile of cholestyramine may have gradually been identified.

## 2 Materials and methods

### 2.1 Data source

This study relied on the open data resources of the US FAERS to conduct a retrospective analysis of adverse events related to cholestyramine. The data covered the period from the first quarter of 2004 to the fourth quarter of 2024, and the inclusion criteria were all ASCII format original report data that labeled cholestyramine as the “primary suspect drug.” This database integrates drug safety information submitted by global medical staff, patients, and pharmaceutical companies through a spontaneous reporting mechanism.

This study utilized publicly available data from the U.S. Food and drug administration adverse event reporting system (FAERS). The data were accessed and used in accordance with the terms and conditions specified by the FAERS database, ensuring compliance with all relevant regulations and guidelines. As the data are de-identified and publicly accessible, no individual patient information was disclosed, and no additional ethical approval was required for this study. The study design and analysis were conducted in a manner that respects patient privacy and confidentiality. The authors declare no conflicts of interest related to the use of FAERS data in this research.

### 2.2 Data processing procedure

**Figure 1:**
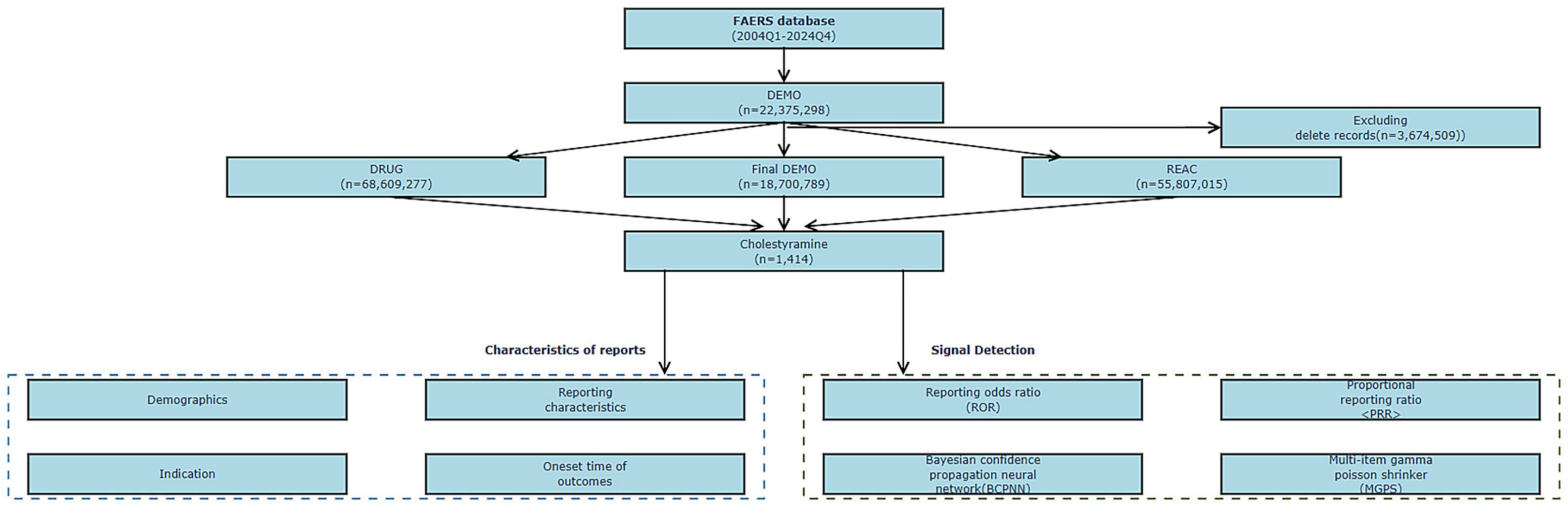
Flowchart demonstrating the AE analysis process for cholestyramine using the FAERS database. AE, adverse event; DEMO, demographic data; DRUG, drug; FAERS, FDA adverse event reporting system; REAC, reaction data; ROR, reporting odds ratio; PRR, proportional reporting ratio; BCPNN, Bayesian confidence propagation neural network; MGPS, multi-item gamma Poisson shrinker; SOC, system organ class; PS, primary suspect; PT, preferred term.

The data cleaning and standardization process included three stages as follows. The first stage implemented the FDA-recommended hierarchical deduplication strategy, which involved sorting by case unique identifier (CASEID) and prioritizing the report with the most recent receipt date (FDA_DT), selecting the record with a larger PRIMARYID value when CASEID and FDA_DT were completely the same, and synchronizing the verification of the deletion list for quarterly data update packages after 2019 to exclude invalid entries. The second stage completed medical coding transformation, based on MedDRA terminology library version 27.1, to uniformly map the original adverse event descriptions to standardized preferred terms (PT) and system organ classification (SOC). The final stage constructed a structured dataset, and the specific processing procedure is shown in Figure 1, the technical roadmap.

### 2.3 Statistical analysis strategy

An incremental analysis framework was adopted: (1) descriptive statistics: to parse the composition characteristics of the dataset from dimensions such as demographic characteristics, report sources, and geographical distribution; (2)signal detection: integrating four internationally accepted disproportionality analysis methods—reporting odds ratio (ROR), proportional reporting ratio (PRR, with thresholds set according to the MHRA standard), Bayesian confidence propagation neural network (BCPNN), and multi-item gamma Poisson shrinkage (MGPS); (3)time series modeling: constructing a Weibull distribution model to explore the temporal dynamic characteristics of adverse events. The analysis process was implemented on the SAS 9.4 platform, and the algorithm parameters and calculation formulas are detailed in Supplementary Tables S1, S2.

An incremental analysis framework was adopted, including descriptive statistics to parse the composition characteristics of the dataset from dimensions such as demographic characteristics, report sources, and geographical distribution; signal detection integrating four internationally accepted disproportionality analysis methods—reporting odds ratio (ROR), proportional reporting ratio (PRR) with thresholds set according to the MHRA standard, Bayesian confidence propagation neural network (BCPNN), and multi-item gamma Poisson shrinkage (MGPS), and time series modeling constructing a Weibull distribution model to explore the temporal dynamic characteristics of adverse events. The analysis process was implemented on the SAS 9.4 platform, and the algorithm parameters and calculation formulas are detailed in Supplementary Tables S1, S2.

## 3 Results

### 3.1 Clinical characteristics

A total of 1,414 adverse event reports with cholestyramine as the primary suspect drug were included in this study (Figure 1). Among them, 62.09% were female patients, 24.11% were male patients, and 13.79% had unknown gender. The age group with the highest proportion was >65 years old (24.05%), followed by the 18–65 years group (18.25%), with the <18 years group being the least (0.5%), and 57.21% had unknown age. Most reports were submitted by consumers (69.87%), doctors (12.94%), pharmacists (8.7%), and other health professionals (5.52%). Adverse events were mainly concentrated in the years 2015–2024 after the drug was marketed, with very few adverse events occurring in 2004–2015, which is considered to be related to the initial lack of widespread application of the drug. 89.89% of the reports came from the United States. Adverse reactions often led to patient hospitalization (22.53%), disability (4.74%), or life-threatening conditions (3.95%). See Table 1 for details.

**Table 1:** Clinical characteristics of AE reports related to cholestyramine from the FAERS database (Q1 2004-Q4 2024).

Characteristics	Number of cases	Proportion of cases (%)
Number of AE reports	1,414	
Sex		
Male	341	24.11
Female	878	62.09
Not specified	195	13.79
Age		
<18	7	0.5
18–65	258	18.25
>65	340	24.05
Not specified	809	57.21
Reporter		
Consumer	988	69.87
health-professor	36	2.55
Other health professional	42	2.97
Pharmacist	123	8.7
Physician	183	12.94
Not specified	42	2.97
Reporting year		
2004	16	1.13
2005	10	0.71
2006	3	0.21
2007	7	0.50
2008	15	1.06
2009	12	0.85
2010	7	0.50
2011	6	0.42
2012	9	0.64
2013	11	0.78
2014	9	0.64
2015	100	7.07
2016	113	7.99
2017	139	9.83
2018	128	9.05
2019	211	14.92
2020	107	7.57
2021	130	9.19
2022	144	10.18
2023	100	7.07
2024	137	9.69
Top 5 reporting countries		
United States of America	1,271	89.89
France	7	0.5
Germany	7	0.5
Great Britain	5	0.35
Denmark	3	0.21
Outcome		
Life-threatening	10	3.95
Hospitalization—initial or prolonged	57	22.53
Disability	12	4.74
Death	5	1.98
Required intervention to prevent permanent impairment/damage	8	3.16
Congenital anomaly	10	3.95
Other	151	59.68

### 3.2 Adverse event distribution at the system organ class (SOC) level

Adverse events related to cholestyramine involved all 27 SOC classifications. As shown in Table 2, significant categories included, but were not limited to gastrointestinal disorders, product issues, injury, poisoning and procedural complications, general disorders and administration site conditions, respiratory, thoracic and mediastinal disorders, investigations, nervous system disorders, musculoskeletal and connective tissue disorders, skin and subcutaneous tissue disorders, metabolism and nutrition disorders, psychiatric disorders, immune system disorders, infections and infestations, and renal and urinary disorders. Table 2 shows the signal strength at the SOC level.

**Table 2:** Signal strength of cholestyramine-related AEs at the system organ class (SOC) level in the FAERS database.

System organ class (SOC)	Case reports	ROR (95% CI)	PRR (95% CI)	IC (IC025)	EBGM (EBGM05)
Gastrointestinal disorders	1,026	3.869	3.191	1.561	3.190
Product issues	795	34.534	28.390	4.655	28.330
Injury, poisoning and procedural complications	713	3.026	2.693	1.301	2.692
General disorders and administration site conditions	534	0.926	0.935	−0.236	0.935
Respiratory, thoracic and mediastinal disorders	336	2.770	2.632	1.220	2.632
Investigations	211	1.429	1.409	0.283	1.409
Nervous system disorders	174	0.727	0.738	−0.664	0.738
Musculoskeletal and connective tissue disorders	101	0.668	0.676	−0.854	0.676
Skin and subcutaneous tissue disorders	99	0.634	0.643	−0.928	0.643
Metabolism and nutrition disorders	45	1.194	1.192	−0.183	1.192
Psychiatric disorders	39	0.374	0.380	−1.835	0.380
Immune system disorders	39	1.262	1.260	−0.137	1.260
Infections and infestations	31	0.260	0.265	−2.395	0.265
Renal and urinary disorders	28	0.661	0.663	−1.116	0.663
Hepatobiliary disorders	20	1.416	1.415	−0.161	1.414
Neoplasms benign, malignant and unspecified (incl cysts and polyps)	14	0.722	0.723	−1.190	0.723
Vascular disorders	13	0.250	0.252	−2.682	0.252
Congenital, familial and genetic disorders	13	11.480	11.449	1.938	11.440
Blood and lymphatic system disorders	12	0.449	0.451	−1.890	0.451
Eye disorders	12	0.434	0.435	−1.939	0.435
Cardiac disorders	11	0.273	0.275	−2.607	0.275
Ear and labyrinth disorders	10	0.802	0.803	−1.164	0.803
Reproductive system and breast disorders	8	1.102	1.102	−0.840	1.102
Surgical and medical procedures	8	0.910	0.910	−1.085	0.910
Pregnancy, puerperium and perinatal conditions	6	0.872	0.873	−1.263	0.873
Social circumstances	2	2.428	2.427	−1.244	1.618
Endocrine disorders	2	0.914	0.915	−2.180	0.610

Ranked by case reports.

### 3.3 Adverse event distribution at the preferred term (PT) level

Adverse events related to cholestyramine were sorted by frequency of occurrence and assessed for signals. Among the adverse events in the FAERS, the side effects mentioned in the package insert are as follows. The most common adverse reaction is constipation, especially in patients with high doses of cholestyramine and those over 60 years old. Less common adverse reactions include abdominal discomfort, bloating, nausea, vomiting, diarrhea, belching, anorexia, steatorrhea, bleeding tendency, night blindness, hyperchloremic acidosis, osteoporosis, rash, and local irritation caused by vitamin K, A, and D deficiency. In children, there have been rare reports of intestinal obstruction (including two deaths), and some patients may experience biliary calcification or biliary colic, but these may be related to liver disease rather than directly caused by the drug. Other reported events (not necessarily related to the drug) include gastrointestinal symptoms (such as rectal bleeding, dysphagia, pancreatitis, etc.), laboratory test abnormalities (liver function abnormalities, prolonged prothrombin time, etc.), allergic reactions (urticaria, asthma, dyspnea, etc.), musculoskeletal pain, nervous system symptoms (headache, anxiety, vertigo, dizziness, fatigue, tinnitus, syncope, somnolence, femoral neuralgia, paresthesia, etc.), and eye and kidney problems (uveitis, hematuria, etc.).

Adverse reactions not mentioned in the package insert found by FAERS analysis include as following. Off-label use, unapproved indications, intentional product misuse, and use of prescription drugs without a prescription. Product-related issues, including issues with product solubility, taste, physical consistency, formulation, packaging, supply, container, and product quality issues, physical contamination of the product, product sedimentation, etc. Newly discovered adverse reactions include discomfort or abnormalities in the respiratory tract, mouth, and throat (foreign body sensation in the mouth, foreign body sensation in the throat, foreign body sensation in the respiratory tract, dysphonia, cough, choking, choking sensation, oropharyngeal pain, tooth discoloration, gastroesophageal reflux disease, irritable bowel syndrome, etc.), fecal abnormalities (color change, softening, hardening), and abnormal urine odor. Others are as follows: blood glucose fluctuations, tooth fracture, exacerbation of concomitant diseases, etc. Table 3 shows the details of the top 50 adverse events at the PT level. All adverse events that meet the positive signal criteria are listed in the Supplementary Table.

**Table 3:** Top 50 most frequent AEs for cholestyramine at the preferred term (PT) level.

Preferred term (PT)	Case reports	ROR (95% CI)	PRR (95% CI)	IC (IC025)	EBGM (EBGM05)
Diarrhoea	183	4.275	4.137	1.802	4.136
Product solubility abnormal	176	562.874	540.083	6.820	518.357
Product taste abnormal	170	194.677	187.089	6.246	184.421
Product use in unapproved indication	159	10.465	10.118	3.021	10.111
Product substitution issue	150	38.401	37.108	4.658	37.004
Product physical consistency issue	126	373.663	362.842	6.284	352.913
Drug ineffective for unapproved indication	94	25.610	25.077	4.017	25.030
Product use complaint	92	101.602	99.469	5.283	98.713
Constipation	85	5.895	5.800	2.139	5.797
Throat irritation	69	22.806	22.459	3.751	22.421
Abdominal pain upper	65	4.622	4.568	1.754	4.566
Abdominal discomfort	62	5.405	5.342	1.951	5.340
Retching	59	40.530	39.993	4.219	39.872
Product use issue	49	3.913	3.880	1.462	3.879
Foreign body in throat	47	270.495	267.576	4.926	262.143
Cough	44	2.290	2.277	0.712	2.277
Choking	42	31.411	31.116	3.747	31.044
Product odour abnormal	42	61.697	61.110	4.224	60.826
Product residue present	41	122.560	121.411	4.519	120.285
Product quality issue	38	3.874	3.849	1.376	3.848
Dysgeusia	36	6.677	6.630	2.045	6.627
Flatulence	33	8.498	8.441	2.292	8.436
Oropharyngeal pain	32	4.967	4.938	1.634	4.937
Blood glucose increased	28	2.070	2.063	0.453	2.063
Choking sensation	28	74.082	73.610	3.851	73.197
Dyspepsia	27	4.004	3.985	1.299	3.985
Abdominal distension	26	3.641	3.625	1.164	3.624
Oropharyngeal discomfort	23	40.256	40.048	3.336	39.927
Product physical issue	22	16.636	16.557	2.698	16.537
Dysphagia	21	3.196	3.185	0.916	3.185
Gastrointestinal disorder	14	2.433	2.429	0.401	2.429
Tooth discolouration	14	65.849	65.640	2.879	65.312
Intentional product use issue	13	2.186	2.182	0.235	2.182
Gastrooesophageal reflux disease	13	2.344	2.340	0.320	2.340
Illness	12	2.082	2.079	0.138	2.079
Drug effective for unapproved indication	12	6.508	6.493	1.387	6.490
Foreign body in mouth	11	2287.538	2281.741	2.741	1938.068
Dysphonia	11	2.696	2.692	0.402	2.692
Product packaging difficult to open	10	70.630	70.470	2.395	70.091
Product availability issue	10	7.280	7.266	1.338	7.262
Rectal haemorrhage	10	3.264	3.258	0.562	3.258
Intestinal obstruction	10	3.965	3.958	0.768	3.957
Throat tightness	10	5.297	5.287	1.055	5.286
Therapeutic response unexpected	10	3.096	3.092	0.504	3.091
Poor quality product administered	10	16.749	16.713	1.910	16.693
Poor quality product administered	10	16.749	16.713	1.910	16.693
Reaction to excipient	9	57.991	57.873	2.198	57.618
Foreign body in respiratory tract	9	90.471	90.286	2.269	89.663
Abnormal faeces	9	15.860	15.830	1.757	15.811
Oral discomfort	9	9.118	9.101	1.415	9.095
Abnormal faeces	9	15.860	15.830	1.757	15.811

### 3.4 Time of adverse event occurrence

**Figure 2:**
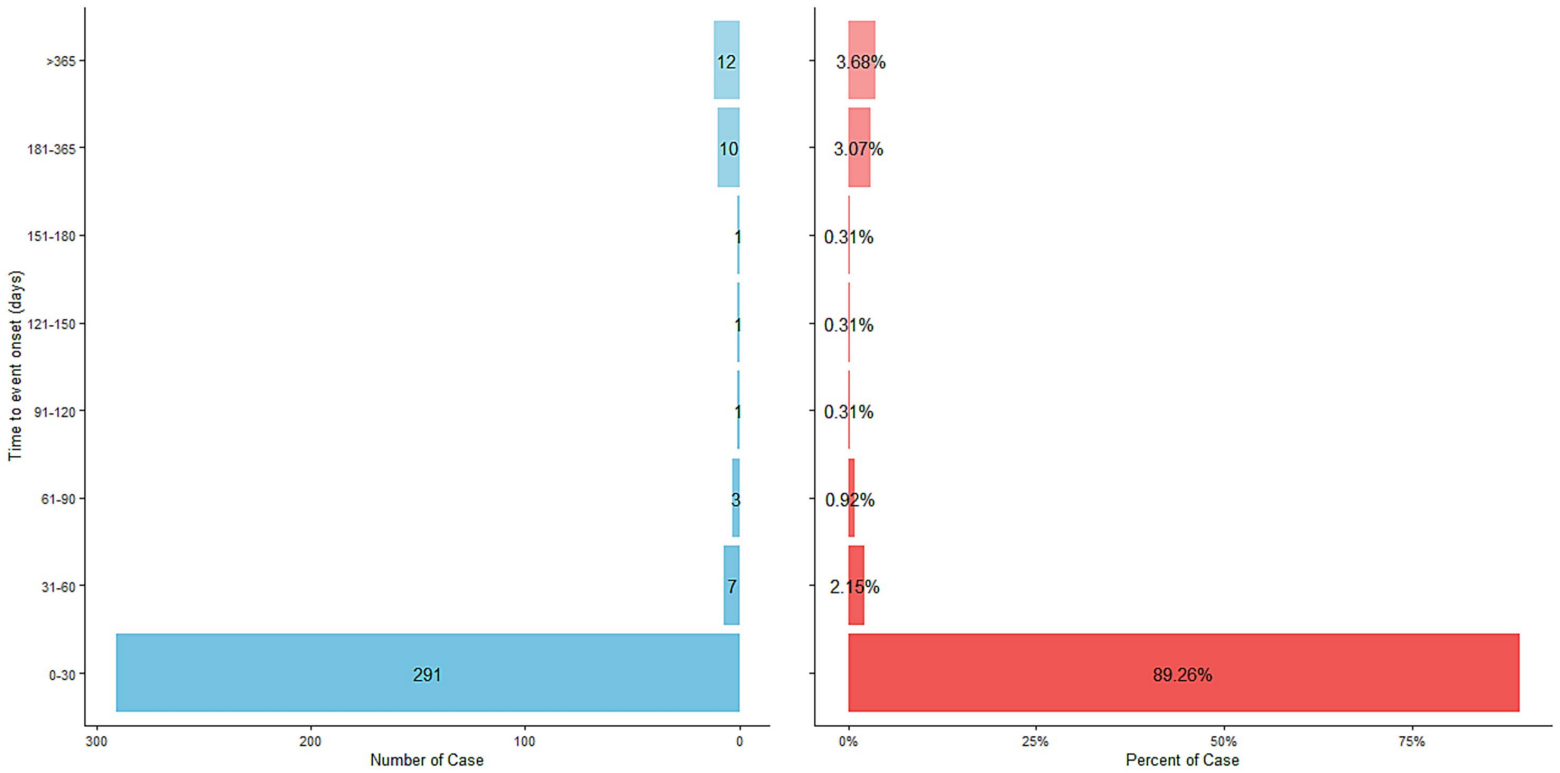
Time to event report distribution of AE reports. AE, adverse event.

**Figure 3:**
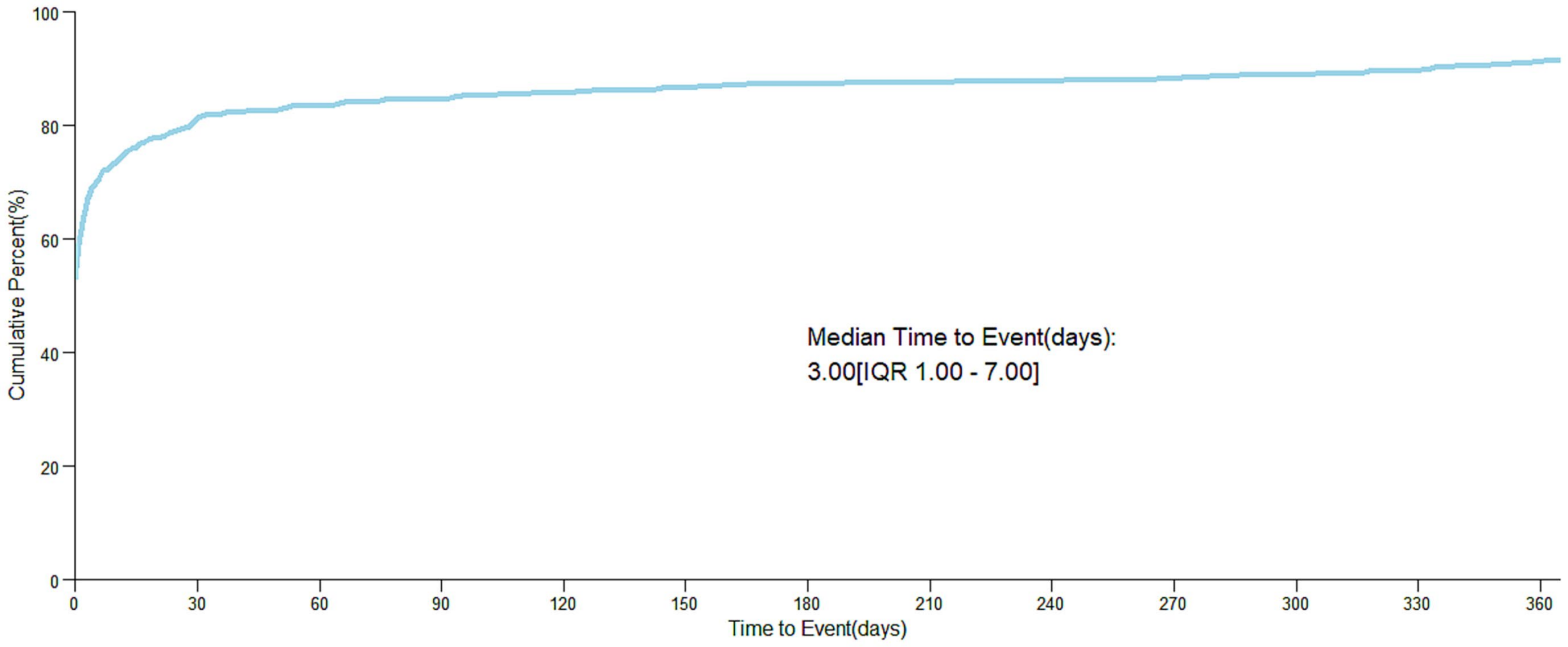
Cumulative incidence of AEs. AE, adverse event.

After excluding reports with false or missing adverse event occurrence times, 326 cholestyramine-related adverse events provided occurrence time data. Most adverse events occurred immediately after administration (*n* = 234, 71.78%) and within 3 days after administration (1 day after administration *n* = 14, 4.29%; 2 days after administration *n* = 6, 1.84%; 3 days after administration *n* = 7, 2.15%), with the highest proportion of adverse reactions occurring within 1 month after administration (*n* = 291, 89.26%). Rare cases occurred 2 months (*n* = 7, 2.15%), 3 months (*n* = 3, 0.92%), 4 months (*n* = 1, 0.31%), 5 months (*n* = 1, 0.31%), 6 months (*n* = 1, 0.31%), 7 to 12 months (*n* = 10, 3.07%), and more than 1 year (*n* = 12, 3.68%) after administration. The median occurrence time was 3 days (interquartile range [IQR] 1.00–7.00 days). The time distribution is shown in Figure 2, and the cumulative incidence curve is shown in Figure 3.

## 4 Discussion

Hypercholesterolemia is a metabolic disorder characterized by elevated blood cholesterol, particularly LDL-C (12). Aetiologically, it is subdivided into primary forms (familial monogenic or polygenic hypercholesterolemia), and secondary forms attributable to diseases (such as hypothyroidism, diabetes, and liver disorders) or drugs (such as glucocorticoids and *β*-blockers) (13). Irrespective of cause, hypercholesterolemia is an independent driver of atherosclerosis, stroke, and CHD, and therefore constitutes a serious human-health threat (14). The therapeutic cornerstoen is to reduce LDL-C levels to attenuate atherosclerosis and cardiovascular diseases. Cholestyramine is a bile acid-binding resin that lowers plasma LDL-C levels by binding bile acids in the intestine and promoting the metabolism of cholesterol to bile acids in cells, and it can be used for patients intolerant to statins (15). Despite its well-documented efficacy safety concerns regarding cholestyramine persist. Through data mining of the FAERS database, this study uncovers potential serious adverse events associated with cholestyramine use, highlighting findings of substantial clinical significance.

This investigation conducted a comprehensive assessment of adverse events related to cholestyramine since its market release in 2004. By analyzing the FAERS database, the study confirmed the common adverse events listed in the drug’s package insert, including constipation, abdominal discomfort, bloating, nausea, vomiting, diarrhea, belching, anorexia, steatorrhea, and bleeding tendencies, night blindness, hyperchloremic acidosis, osteoporosis, rashes, and local irritation caused by deficiencies in vitamins K, A, and D. Additionally, potential adverse events not listed in the package insert were identified, such as off-label use, unapproved indications, discomfort or abnormalities in the respiratory tract, mouth, and throat, gastroesophageal reflux disease (GERD), irritable bowel syndrome (IBS), fecal abnormalities (color changes, softening, hardening), blood glucose fluctuations, tooth fracture, and exacerbation of concomitant diseases. These findings highlight the importance of strengthening drug monitoring, especially during the initial administration of cholestyramine, to effectively manage potential side effects.

It is noteworthy that the formulation of cholestyramine—a powder requires reconstitution before administration—may increase the risk of medication errors. Data from the FAERS indicate that the incidence of esophageal obstruction or mucosal injury is significantly higher in patients who swallow the drug directly without dissolving it compared to other formulations. Off-label use, identified as a potential risk factor for adverse reactions associated with cholestyramine, refer to clinical prescribing practices that extend beyond the approved indications, target populations, dosages, or target populations approved by drug regulatory authorities in the drug’s package insert (16). Currently, off-label use is prevalent in clinical practice for several reasons. Firstly, the absence of targeted therapies for certain diseases, such as rare diseases, prompts doctors to explore new therapeutic applications for existing drugs. Secondly, although emerging evidence may support the efficacy of a drug for a new indication, pharmaceutical companies may not have pursued regulatory approval for that use. Thirdly, the need for individualized treatment arises when patients are intolerant to conventional treatments and require dosage adjustments. Lastly, cost considerations lead patients to choose cheaper old drugs over expensive new ones due to economic issues (17). Thus, it appears that appropriate explanations are: when using cholestyramine, doctors or pharmacists may not clearly instruct patients, and patients may not use cholestyramine according to the required indications, dosages, etc., in the package insert, leading to off-label use. To mitigate these risks, clinicians should adhere to standardized prescribing protocols and reinforce patient education regarding proper medication handling and potential adverse effects.

GERD is another potential adverse reaction. GERD refers to a condition where the contents of the duodenum or stomach reflux into the esophagus, mouth, or airway, causing reflux esophagitis or damage to extra-esophageal tissues (18). Common manifestations of GERD include persistent cough, heartburn, acid regurgitation, and chest pain. The pathogenesis of GERD is highly complex and may involve the direct corrosive effects of stomach acid and pepsin on the esophageal mucosa, vagal nerve dysfunction, inflammatory responses, increased esophageal sensitivity, and *Helicobacter pylori* infection (19). Known adverse reactions of cholestyramine include nausea and vomiting (20). Thus, a possible explanation is that cholestyramine use, which causes nausea and vomiting, leads to the reflux of stomach contents, including stomach acid and pepsin, into the esophagus, directly corroding the esophageal mucosa and causing esophageal inflammation, erosion, and even ulcers. The relationship between cholestyramine and GERD needs further research to be proven, but this adverse reaction provides a basis for doctors to prevent GERD when using cholestyramine in patients with hypercholesterolemia.

IBS has been identified as a potential adverse reaction associated with cholestyramine use. IBS is a functional gastrointestinal disorder characterized by symptoms such as abdominal pain, bloating, and changes in bowel habits (21). Its current incidence ranges from 2 to 12%, though actual rates are likely higher, with a slightly predominance in females. IBS is typically classified into four subtypes: constipation-predominant, diarrhea-predominant, mixed, and unsubtyped IBS (22). The condition is often recurrent and chronic, significantly affecting patients’ quality of life. Studies indicate that IBS arises from a multifactorial interplay of elements, including psychological disorders, gastrointestinal motility abnormalities, gastrointestinal infections and inflammation, visceral hypersensitivity, and gastrointestinal hormones (23). These underlying mechanisms align with several documented adverse reactions of cholestyramine, such as constipation, abdominal discomfort, and diarrhea. Thus, a plausible explanatory mechanism is that cholestyramine induced constipation, abdominal discomfort, or diarrhea may disrupt gastrointestinal motility, visceral hypersensitivity, exacerbate intestinal infections or inflammation, and alter gastrointestinal hormone secretion, collectively contributing to the occurrence of IBS. During treatment with cholestyramine, close monitoring of intra-abdominal pressure, bowel movements, and other gastrointestinal parameters is recommended. If symptoms such as increased intra-abdominal pressure, abdominal pain, diarrhea, or constipation occur, IBS should be considered in the differential diagnosis. Physicians should advise patients to remain vigilant regarding these potential adverse reactions when prescribing cholestyramine.

Our study also suggests that fecal abnormalities (color changes, softening, hardening) are potential risks associated with cholestyramine. Fecal abnormalities indicate issues with the digestive system or other problems and are commonly manifested as: abnormal color, such as tarry stools indicating upper gastrointestinal bleeding or iron use, bright red stools indicating lower gastrointestinal bleeding such as anal fissures or colorectal cancer, and grayish-white stools possibly caused by biliary obstruction; changes in consistency, such as watery stools indicating infection, food poisoning, or allergies, and hard stools indicating constipation or intestinal obstruction, steatorrhea suggesting pancreatitis (24, 25). Fecal abnormalities are often accompanied by symptoms such as abdominal pain, diarrhea, fever, and weight loss. This corresponds with the known adverse reactions of cholestyramine, such as diarrhea and steatorrhea (26). A plausible explanation is that cholestyramine use, which binds bile acids in the intestines, leads to fatty acid metabolic disturbances and poor fat absorption, causing watery stools and steatorrhea. The relationship between cholestyramine and fecal abnormalities needs further research to be confirmed, but this adverse reaction provides a basis for doctors to monitor fecal tests in patients treated with cholestyramine.

Blood glucose fluctuations are potential adverse reactions identified in our study. Blood glucose fluctuations, also known as glycemic variability, refer to the unstable state of blood glucose levels fluctuating between high and low values at different time intervals (27). Physiological blood glucose fluctuations occur under the regulation of the neuroendocrine system to adapt to environmental changes, with a general fluctuation range of 2–3 mmol/L within 24 h (28). Patients, due to infections, stress, and other factors, may experience pancreatic cell damage, increased insulin resistance, and increased secretion of glucagon and other hormones, resulting in more significant blood glucose levels and fluctuation amplitudes compared to normal individuals (29). The cause of blood glucose fluctuations induced by cholestyramine may be that cholestyramine binds to bile acids in the intestines, indirectly affecting fat metabolism and intestinal hormones, impacting insulin sensitivity. Cholestyramine may affect the absorption of other hypoglycemic drugs such as metformin, influencing blood glucose fluctuations. Further research is needed to explore the relationship between cholestyramine and blood glucose fluctuations. In summary, blood glucose fluctuations are potential adverse reactions identified in our study. When using cholestyramine, closely monitor blood glucose changes and provide timely symptomatic treatment.

Tooth fracture has been identified as a potential adverse reaction associated with cholestyramine. Tooth fracture refers to the transverse or longitudinal splitting of teeth caused by trauma, biting hard objects, or weakened tooth structure (30). The organic content in teeth decreases with age, while the inorganic content gradually increases, causing teeth to become more brittle; after biting hard objects, enamel damage can lead to tooth fractures (31). This corresponds with the known adverse reactions of cholestyramine, such as vitamin D deficiency and osteoporosis. A plausible explanation is that cholestyramine use, which causes vitamin D deficiency and osteoporosis, leads to thinner and more fragile bone tissue, making teeth more susceptible to damage or fractures; severe osteoporosis can cause thinning of the alveolar bone, leading to loose teeth, receding gums, and tooth loss. This adverse reaction provides a basis for doctors to advise patients to be cautious about such adverse reactions when treating them with cholestyramine for hypercholesterolemia.

Exacerbation of concomitant diseases is a potential adverse reaction identified in our study. Exacerbation of concomitant diseases refers to the increased risk of worsening conditions and symptom intensification when multiple diseases coexist in a patient, commonly seen in elderly patients, those with chronic illnesses, and individuals with weakened immune systems. The causes of exacerbation of concomitant diseases may include: interactions between different diseases leading to complex conditions, decreased immunity making patients more susceptible to infections, and interactions between different medications affecting drug efficacy (32). The cause of exacerbation of concomitant diseases induced by cholestyramine may be: cholestyramine binding to bile acids in the intestines indirectly affects fat metabolism and the secretion of intestinal hormones, reducing immunity; cholestyramine use, which causes deficiencies in vitamins K, A, and D, leading to bleeding tendencies, may exacerbate damage to other organs. In summary, exacerbation of concomitant diseases is a potential adverse reaction identified in our study. When treating patients with cholestyramine, doctors should advise patients to be cautious about such adverse reactions.

This study conducted a disproportionality analysis of the FAERS data and predicted the cumulative incidence and timing of adverse reactions associated with cholestyramine as the primary suspect drug. This indicates the need for early detection and treatment of related adverse reactions following cholestyramine treatment for hypercholesterolemia and other diseases. This provides strong evidence for the prevention and treatment of adverse reactions, which is beneficial for reducing adverse reactions and improving disease prognosis.

The study has certain limitations. Firstly, the FAERS database is a reporting system provided by patients, pharmacists, and doctors, and there may be inaccuracies and missing data. We have implemented a rigorous data processing procedure, including correcting obvious data entry errors and deleting duplicate reports, to increase the sample size and offset the impact of individual inaccurate data, making our conclusions more accurate. Secondly, our data are all from the United States, so the conclusions are more biased towards North America and may have deviations for regions in Asia, Africa, and Latin America. Lastly, while the FAERS database can provide potential risks of adverse reactions, it offers limited assistance with patient epidemiological data.

## 5 Conclusion

This study utilized FAERS data to conduct a disproportionality analysis of adverse events associated with cholestyramine, focusing on all reports from the first quarter of 2004 to the fourth quarter of 2024. The analysis results confirmed known adverse reactions of cholestyramine, such as constipation, abdominal discomfort, bloating, nausea, vomiting, diarrhea, belching, anorexia, steatorrhea, and bleeding tendencies, night blindness, hyperchloremic acidosis, osteoporosis, rashes, and local irritation caused by deficiencies in vitamins K, A, and D. It also identified potential adverse reactions such as off-label use, unapproved indications, discomfort or abnormalities in the respiratory tract, mouth, and throat, GERD, IBS, fecal abnormalities (color changes, softening, hardening), blood glucose fluctuations, tooth fracture, and exacerbation of concomitant diseases. These findings underscore the necessity of early monitoring of adverse reactions in patients to ensure the safe application of cholestyramine in the treatment of hypercholesterolemia and other diseases.

## Data Availability

The original contributions presented in the study are included in the article/Supplementary material, further inquiries can be directed to the corresponding author.

## References

[1] Groenendyk JW Greenland P Khan SS. Incremental value of polygenic risk scores in primary prevention of coronary heart disease: a review. JAMA Intern Med. (2022) 182:1082–8. doi: 10.1001/jamainternmed.2022.3171, 35994254

[2] Shaya GE Leucker TM Jones SR Martin SS Toth PP. Coronary heart disease risk: low-density lipoprotein and beyond. Trends Cardiovasc Med. (2022) 32:181–94. doi: 10.1016/j.tcm.2021.04.002, 33872757

[3] Bjornson E Adiels M Taskinen MR Burgess S Chapman MJ Packard CJ . Lipoprotein(a) is markedly more atherogenic than LDL: an apolipoprotein B-based genetic analysis. J Am Coll Cardiol. (2024) 83:385–95. doi: 10.1016/j.jacc.2023.10.039, 38233012 PMC7616706

[4] Macchiaiolo M Gagliardi MG Toscano A Guccione P Bartuli A. Homozygous familial hypercholesterolaemia. Lancet. (2012) 379:1330. doi: 10.1016/S0140-6736(11)61476-1 22285056

[5] Sjoberg BG Straniero S Angelin B Rudling M. Cholestyramine treatment of healthy humans rapidly induces transient hypertriglyceridemia when treatment is initiated. Am J Physiol Endocrinol Metab. (2017) 313:E167–74. doi: 10.1152/ajpendo.00416.2016, 28487440

[6] Beuers U Wolters F Oude Elferink RPJ. Mechanisms of pruritus in cholestasis: understanding and treating the itch. Nat Rev Gastroenterol Hepatol. (2023) 20:26–36. doi: 10.1038/s41575-022-00687-7, 36307649

[7] Scaldaferri F Pizzoferrato M Ponziani FR Gasbarrini G Gasbarrini A. Use and indications of cholestyramine and bile acid sequestrants. Intern Emerg Med. (2013) 8:205–10. doi: 10.1007/s11739-011-0653-0, 21739227

[8] Selim R Ahn J. Pruritus in chronic liver disease. Clin Liver Dis. (2023) 27:47–55. doi: 10.1016/j.cld.2022.08.011 36400466

[9] Hutcheon DF Bayless TM Gadacz TR. Postcholecystectomy diarrhea. JAMA. (1979) 241:823–4. doi: 10.1001/jama.1979.03290340041024 762849

[10] Muchiri RN Rocha J Tandon A Chen YL Alemani R Ahmad I . Short-term treatment with cholestyramine increases long-acting anticoagulant rodenticide clearance from rabbits without affecting plasma vitamin K1 levels or blood coagulation. Toxicol Sci. (2024) 200:137–45. doi: 10.1093/toxsci/kfae053, 38603617 PMC11199916

[11] Xu X Guo Q Li Y Zhai C Mao Y Zhang Y . Assessing the real-world safety of regadenoson for myocardial perfusion imaging: insights from a comprehensive analysis of FAERS data. J Clin Med. (2025) 14:1860. doi: 10.3390/jcm14061860, 40142667 PMC11943247

[12] Ray KK Bays HE Catapano AL Lalwani ND Bloedon LT Sterling LR . Safety and efficacy of bempedoic acid to reduce LDL cholesterol. N Engl J Med. (2019) 380:1022–32. doi: 10.1056/NEJMoa1803917, 30865796

[13] Lamb YN. Rosuvastatin/ezetimibe: a review in hypercholesterolemia. Am J Cardiovasc Drugs. (2020) 20:381–92. doi: 10.1007/s40256-020-00421-1, 32648167

[14] Stehbens WE. Coronary heart disease, hypercholesterolemia, and atherosclerosis. II. Misrepresented data. Exp Mol Pathol. (2001) 70:120–39. doi: 10.1006/exmp.2000.2339, 11263955

[15] Gershkovich P Sivak O Contreras-Whitney S Darlington JW Wasan KM. Assessment of cholesterol absorption inhibitors nanostructured aluminosilicate and cholestyramine using in vitro lipolysis model. J Pharm Sci. (2012) 101:291–300. doi: 10.1002/jps.22770, 21935955

[16] Lerose R Musto P Aieta M Papa C Tartarone A. Off-label use of anti-cancer drugs between clinical practice and research: the Italian experience. Eur J Clin Pharmacol. (2012) 68:505–12. doi: 10.1007/s00228-011-1173-6 22166932

[17] Saiyed MM Ong PS Chew L. Off-label drug use in oncology: a systematic review of literature. J Clin Pharm Ther. (2017) 42:251–8. doi: 10.1111/jcpt.12507, 28164359

[18] Fass R Boeckxstaens GE El-Serag H Rosen R Sifrim D Vaezi MF. Gastro-oesophageal reflux disease. Nat Rev Dis Primers. (2021) 7:55. doi: 10.1038/s41572-021-00287-w, 34326345

[19] Argüero J Sifrim D. Pathophysiology of gastro-oesophageal reflux disease: implications for diagnosis and management. Nat Rev Gastroenterol Hepatol. (2024) 21:282–93. doi: 10.1038/s41575-023-00883-z 38177402

[20] Panahi Y Khedmat H Valizadegan G Mohtashami R Sahebkar A. Efficacy and safety of *Aloe vera* syrup for the treatment of gastroesophageal reflux disease: a pilot randomized positive-controlled trial. J Tradit Chin Med. (2015) 35:632–6. doi: 10.1016/s0254-6272(15)30151-5, 26742306

[21] Chey WD Kurlander J Eswaran S. Irritable bowel syndrome: a clinical review. JAMA. (2015) 313:949–58. doi: 10.1001/jama.2015.0954, 25734736

[22] Bonetto S Fagoonee S Battaglia E Grassini M Saracco GM Pellicano R. Recent advances in the treatment of irritable bowel syndrome. Pol Arch Intern Med. (2021) 131:709–15. doi: 10.20452/pamw.16067, 34463082

[23] Di Rosa C Altomare A Terrigno V Carbone F Tack J Cicala M . Constipation-predominant irritable bowel syndrome (IBS-C): effects of different nutritional patterns on intestinal dysbiosis and symptoms. Nutrients. (2023) 15:1647. doi: 10.3390/nu15071647 37049488, 37049488 PMC10096616

[24] Patkova B Wester T. Anal fissure in children. Eur J Pediatr Surg. (2020) 30:391–4. doi: 10.1055/s-0040-1716723 32920798

[25] Baunwall SMD Andreasen SE Hansen MM Kelsen J Høyer KL Rågård N . Faecal microbiota transplantation for first or second Clostridioides difficile infection (EarlyFMT): a randomised, double-blind, placebo-controlled trial. Lancet Gastroenterol Hepatol. (2022) 7:1083–91. doi: 10.1016/s2468-1253(22)00276-x, 36152636

[26] Radovanovic-Dinic B Tesic-Rajkovic S Grgov S Petrovic G Zivkovic V. Irritable bowel syndrome—from etiopathogenesis to therapy. Biomed Pap Med Fac Univ Palacky Olomouc Czech Repub. (2018) 162:1–9. doi: 10.5507/bp.2017.057, 29358788

[27] Tang M Kalim S. Long-term glycemic variability: a variable glycemic metric entangled with glycated hemoglobin. Am J Kidney Dis. (2023) 82:254–6. doi: 10.1053/j.ajkd.2023.06.001, 37389509

[28] Silver B Ramaiya K Andrew SB Fredrick O Bajaj S Kalra S . EADSG guidelines: insulin therapy in diabetes. Diabetes Ther. (2018) 9:449–92. doi: 10.1007/s13300-018-0384-6, 29508275 PMC6104264

[29] Hoang K Ly A Hill D. Effect of glycemic variability on infectious outcomes in critically ill burn patients. Burns J Int Soc Burn Inj. (2024) 50:1555–61. doi: 10.1016/j.burns.2024.03.037, 38604824

[30] Marasca B Ndokaj A Duś-Ilnicka I Nisii A Marasca R Bossù M . Management of transverse root fractures in dental trauma. Dent Med Probl. (2022) 59:637–45. doi: 10.17219/dmp/145895, 36537854

[31] Ziscovici C Lucas PW Constantino PJ Bromage TG van Casteren A. Sea otter dental enamel is highly resistant to chipping due to its microstructure. Biol Lett. (2014) 10:20140484. doi: 10.1098/rsbl.2014.0484, 25319817 PMC4272202

[32] Sunderland KM Beaton D Arnott SR Kleinstiver P Binns MA. The ontario neurodegenerative disease research initiative. Cold Spring Harbor, NY: Cold Spring Harbor Laboratory Press (2020).

