# Supplementary material for: Evaluating the real-world safety of cholestyramine for the treatment of hyperlipidemia: disproportionality analysis of FAERS data
Source: Front Med (Lausanne). 2026 Feb 11;13:1765949. doi: 10.3389/fmed.2026.1765949 (PMC12932148; doi:10.3389/fmed.2026.1765949)
Supplement: Supplementary file 1 [file Supplementary_file_1.docx]

Supplementary Material

Supplementary Tables

Supplementary Table 1: Two-by-two contingency table for disproportionality analyses

|  | Target AEs | Other AEs | Total |
| --- | --- | --- | --- |
| eptifibatide | a | b | a+b |
| Other drugs | c | d | c+d |
| Total | a+c | c+d | a+b+c+d |

Abbreviations: AEs, adverse events; a, the number of reports containing target AEs caused by Eptifibatide; b, the number of reports containing other AEs caused by Eptifibatide; c, the number of reports containing target AEs caused by other drugs; b, the number of reports containing other AEs caused by other drugs.

Supplementary Table 2: Five major algorithms used for signal detection.

| eptifibatide | Equation | Criteria |
| --- | --- | --- |
| ROR | 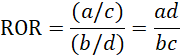  95%CI=e^ln(ROR)±1.96(1/a+1/b+1/c+1/d)^0.5^ | N≥3 and the 95% CI lower limit>1 |
| PRR | 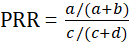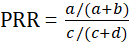  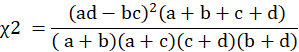 | PRR (95% CI): N≥3 and the 95% CI lower limit>1  PRR (χ^2^): N≥3, PRR≥2 and χ^2^≥4 |
| BCPNN | IC=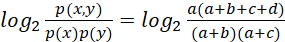  E(IC)=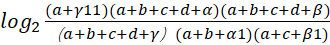  V(IC)=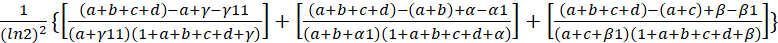  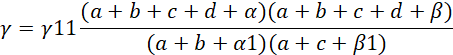  IC-2SD=E(IC)-2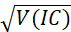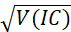  α1=β1, α=β=2 | Lower limit of IC025>0 |
| MGPS | 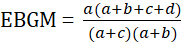  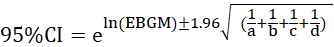 | EBGM05>2 |

Abbreviations: AEs, adverse events; a, the number of reports containing target AEs caused by eptifibatide; b, the number of reports containing other AEs caused by eptifibatide; c, the number of reports containing target AEs caused by other drugs; b, the number of reports containing other AEs caused by other drugs; CI, confidence interval; N, the number of reports; χ^2^, chi-squared; IC, information component; IC025, the lower limit of 95% CI of the IC; E(IC), the IC expectations; V(IC), the variance of IC; EBGM, empirical Bayesian geometric mean; EBGM05, the lower limit of 95% CI of EBGM.

Supplementary Table 3 Signal strength of AEs at the Preferred Term (PT) level ranked by FAERS data.

| Preferred Term (PT) | Case reports | ROR (95% CI) | PRR (95% CI) | PRR (χ^2^) | IC (IC025) | EBGM (EBGM05) |
| --- | --- | --- | --- | --- | --- | --- |
| Thrombocytopenia | 264 | 49.51 (43.65,56.15) | 45.56 (40.58,51.15) | 11495.6 | 5.51 (5.10) | 45.44 (40.06) |
| Haemorrhage | 92 | 17.43 (14.16,21.45) | 16.96 (13.87,20.75) | 1383.04 | 4.08 (3.55) | 16.95 (13.77) |
| Platelet count decreased | 74 | 13.44 (10.67,16.92) | 13.15 (10.50,16.48) | 831.73 | 3.72 (3.16) | 13.14 (10.44) |
| Post procedural complication | 61 | 60.08 (46.61,77.43) | 58.97 (45.97,75.64) | 3465.24 | 5.88 (4.56) | 58.77 (45.60) |
| Product use in unapproved indication | 58 | 5.01 (3.86,6.49) | 4.94 (3.82,6.37) | 182.64 | 2.30 (1.83) | 4.93 (3.81) |
| Retroperitoneal haemorrhage | 54 | 407.26 (310.26,534.59) | 400.50 (306.46,523.40) | 21026.4 | 8.61 (5.20) | 391.34 (298.12) |
| Hypotension | 53 | 5.08 (3.87,6.67) | 5.02 (3.84,6.55) | 170.96 | 2.33 (1.83) | 5.02 (3.82) |
| Gastrointestinal haemorrhage | 49 | 10.77 (8.12,14.28) | 10.62 (8.04,14.03) | 427.41 | 3.41 (2.74) | 10.62 (8.01) |
| Haemorrhage intracranial | 48 | 59.27 (44.55,78.85) | 58.41 (44.09,77.38) | 2699.77 | 5.86 (4.33) | 58.21 (43.75) |
| Haemoglobin decreased | 42 | 7.69 (5.67,10.43) | 7.61 (5.63,10.27) | 241.27 | 2.93 (2.28) | 7.60 (5.61) |
| Cerebral haemorrhage | 38 | 20.19 (14.66,27.81) | 19.97 (14.55,27.39) | 684.21 | 4.32 (3.28) | 19.94 (14.48) |
| Haematoma | 36 | 25.81 (18.58,35.85) | 25.53 (18.45,35.34) | 847.71 | 4.67 (3.46) | 25.50 (18.35) |
| Post procedural haemorrhage | 35 | 56.40 (40.40,78.74) | 55.80 (40.12,77.62) | 1877.97 | 5.80 (3.98) | 55.62 (39.84) |
| Epistaxis | 31 | 7.88 (5.53,11.23) | 7.82 (5.51,11.10) | 184.43 | 2.97 (2.17) | 7.81 (5.49) |
| Pulmonary alveolar haemorrhage | 30 | 107.00 (74.60,153.46) | 106.02 (74.17,151.55) | 3101.82 | 6.72 (4.07) | 105.37 (73.47) |
| Pulmonary haemorrhage | 29 | 68.71 (47.63,99.10) | 68.10 (47.37,97.91) | 1910.00 | 6.08 (3.86) | 67.83 (47.03) |
| Thrombosis | 28 | 6.56 (4.52,9.52) | 6.51 (4.50,9.42) | 130.78 | 2.70 (1.91) | 6.51 (4.49) |
| Haemoptysis | 26 | 17.58 (11.95,25.87) | 17.45 (11.90,25.59) | 402.95 | 4.12 (2.88) | 17.43 (11.85) |
| Blood creatine phosphokinase MB increased | 25 | 346.02 (232.54,514.88) | 343.36 (231.45,509.39) | 8365.96 | 8.39 (4.02) | 336.61 (226.22) |
| Procedural complication | 25 | 59.60 (40.18,88.40) | 59.15 (40.00,87.46) | 1424.37 | 5.88 (3.62) | 58.95 (39.74) |
| Cardiac arrest | 24 | 5.45 (3.65,8.14) | 5.42 (3.64,8.07) | 86.50 | 2.44 (1.62) | 5.41 (3.62) |
| Haematuria | 24 | 13.04 (8.72,19.48) | 12.95 (8.69,19.29) | 264.59 | 3.69 (2.55) | 12.94 (8.66) |
| Haematemesis | 23 | 17.02 (11.29,25.66) | 16.91 (11.25,25.41) | 344.04 | 4.08 (2.75) | 16.89 (11.21) |
| Petechiae | 23 | 42.46 (28.16,64.03) | 42.17 (28.05,63.40) | 922.31 | 5.39 (3.36) | 42.07 (27.90) |
| Acute myocardial infarction | 22 | 13.64 (8.96,20.74) | 13.55 (8.93,20.55) | 255.65 | 3.76 (2.53) | 13.54 (8.90) |
| Cardiogenic shock | 22 | 30.84 (20.27,46.91) | 30.63 (20.19,46.47) | 629.65 | 4.93 (3.14) | 30.58 (20.10) |
| Bradycardia | 21 | 7.37 (4.80,11.32) | 7.33 (4.78,11.22) | 114.76 | 2.87 (1.89) | 7.32 (4.77) |
| Cardio-respiratory arrest | 20 | 8.77 (5.65,13.61) | 8.72 (5.63,13.50) | 136.69 | 3.12 (2.04) | 8.71 (5.61) |
| Catheter site haemorrhage | 20 | 158.48 (101.90,246.48) | 157.51 (101.55,244.31) | 3081.99 | 7.29 (3.58) | 156.08 (100.36) |
| Ecchymosis | 19 | 51.40 (32.72,80.74) | 51.10 (32.62,80.07) | 930.69 | 5.67 (3.22) | 50.96 (32.44) |
| Post procedural haematoma | 19 | 206.33 (131.08,324.78) | 205.13 (130.66,322.05) | 3813.84 | 7.66 (3.54) | 202.71 (128.78) |
| Medication error | 17 | 5.89 (3.66,9.49) | 5.87 (3.65,9.43) | 68.69 | 2.55 (1.52) | 5.87 (3.64) |
| Vascular stent thrombosis | 16 | 152.04 (92.83,249.01) | 151.29 (92.60,247.20) | 2367.87 | 7.23 (3.24) | 149.97 (91.57) |
| Cardiac tamponade | 15 | 59.65 (35.89,99.15) | 59.38 (35.81,98.47) | 858.05 | 5.89 (2.95) | 59.18 (35.60) |
| Coronary artery thrombosis | 15 | 134.87 (81.05,224.42) | 134.25 (80.87,222.87) | 1968.39 | 7.06 (3.12) | 133.21 (80.05) |
| Wound secretion | 15 | 71.36 (42.93,118.64) | 71.04 (42.83,117.82) | 1031.54 | 6.14 (3.00) | 70.75 (42.56) |
| Hypoxia | 14 | 7.73 (4.57,13.06) | 7.70 (4.56,12.98) | 81.60 | 2.94 (1.66) | 7.70 (4.55) |
| Melaena | 14 | 11.84 (7.00,20.02) | 11.80 (6.99,19.90) | 138.28 | 3.56 (2.03) | 11.79 (6.97) |
| Haematocrit decreased | 13 | 11.91 (6.91,20.53) | 11.86 (6.90,20.41) | 129.29 | 3.57 (1.97) | 11.86 (6.88) |
| Subarachnoid haemorrhage | 13 | 23.86 (13.84,41.16) | 23.77 (13.81,40.91) | 283.25 | 4.57 (2.40) | 23.74 (13.77) |
| Disseminated intravascular coagulation | 12 | 15.77 (8.94,27.80) | 15.71 (8.93,27.65) | 165.20 | 3.97 (2.08) | 15.70 (8.90) |
| Gingival bleeding | 12 | 16.64 (9.44,29.35) | 16.59 (9.43,29.18) | 175.64 | 4.05 (2.11) | 16.57 (9.40) |
| Product administration error | 12 | 4.34 (2.46,7.65) | 4.33 (2.46,7.62) | 30.74 | 2.11 (0.98) | 4.33 (2.46) |
| Anaphylactic reaction | 11 | 3.98 (2.20,7.20) | 3.97 (2.20,7.17) | 24.49 | 1.99 (0.84) | 3.97 (2.20) |
| Coronary artery occlusion | 11 | 14.48 (8.01,26.19) | 14.44 (8.00,26.05) | 137.50 | 3.85 (1.93) | 14.43 (7.98) |
| Immune thrombocytopenia | 11 | 23.06 (12.75,41.70) | 22.98 (12.74,41.48) | 231.03 | 4.52 (2.18) | 22.95 (12.69) |
| Coronary artery stenosis | 10 | 35.70 (19.18,66.47) | 35.60 (19.16,66.14) | 335.58 | 5.15 (2.23) | 35.53 (19.08) |
| Upper gastrointestinal haemorrhage | 10 | 11.02 (5.92,20.50) | 10.99 (5.92,20.40) | 90.75 | 3.46 (1.65) | 10.98 (5.90) |
| Ventricular tachycardia | 10 | 11.43 (6.14,21.27) | 11.40 (6.14,21.17) | 94.84 | 3.51 (1.68) | 11.39 (6.12) |
| Vascular pseudoaneurysm | 10 | 110.75 (59.41,206.44) | 110.41 (59.34,205.42) | 1077.25 | 6.78 (2.46) | 109.71 (58.85) |
| Multiple organ dysfunction syndrome | 10 | 4.24 (2.28,7.89) | 4.23 (2.28,7.86) | 24.69 | 2.08 (0.84) | 4.23 (2.27) |
| Pericardial haemorrhage | 9 | 75.76 (39.33,145.95) | 75.55 (39.29,145.29) | 659.21 | 6.23 (2.24) | 75.22 (39.05) |
| Shock | 9 | 7.66 (3.98,14.73) | 7.64 (3.98,14.67) | 51.91 | 2.93 (1.28) | 7.63 (3.97) |
| Coronary artery disease | 8 | 4.63 (2.31,9.26) | 4.62 (2.31,9.23) | 22.70 | 2.21 (0.76) | 4.62 (2.31) |
| Gastric haemorrhage | 8 | 12.60 (6.29,25.22) | 12.57 (6.29,25.12) | 85.15 | 3.65 (1.50) | 12.56 (6.27) |
| Haemorrhagic stroke | 8 | 18.97 (9.48,37.98) | 18.93 (9.47,37.83) | 135.71 | 4.24 (1.70) | 18.91 (9.44) |
| Cardiac procedure complication | 8 | 236.27 (117.50,475.12) | 235.69 (117.41,473.15) | 1844.12 | 7.86 (2.15) | 232.49 (115.62) |
| Electrocardiogram ST segment elevation | 7 | 38.96 (18.54,81.87) | 38.88 (18.54,81.56) | 257.78 | 5.28 (1.74) | 38.80 (18.46) |
| Extravasation | 7 | 31.61 (15.05,66.41) | 31.55 (15.04,66.17) | 206.69 | 4.98 (1.69) | 31.49 (14.99) |
| Acute coronary syndrome | 7 | 15.31 (7.29,32.15) | 15.28 (7.29,32.03) | 93.33 | 3.93 (1.43) | 15.26 (7.27) |
| Catheter site haematoma | 7 | 467.44 (220.44,991.22) | 466.44 (220.31,987.53) | 3164.57 | 8.83 (1.94) | 454.05 (214.12) |
| Thrombosis in device | 7 | 37.51 (17.85,78.81) | 37.43 (17.84,78.52) | 247.68 | 5.22 (1.73) | 37.35 (17.78) |
| Activated partial thromboplastin time prolonged | 6 | 21.41 (9.61,47.72) | 21.37 (9.60,47.56) | 116.38 | 4.42 (1.36) | 21.35 (9.58) |
| Brain herniation | 6 | 38.60 (17.31,86.07) | 38.54 (17.31,85.79) | 218.88 | 5.26 (1.50) | 38.45 (17.25) |
| Coagulation time prolonged | 6 | 71.16 (31.89,158.77) | 71.03 (31.88,158.25) | 412.54 | 6.14 (1.60) | 70.74 (31.70) |
| Haemarthrosis | 6 | 13.76 (6.18,30.67) | 13.74 (6.18,30.57) | 70.83 | 3.78 (1.19) | 13.73 (6.16) |
| Lung infiltration | 6 | 12.01 (5.39,26.77) | 11.99 (5.39,26.69) | 60.43 | 3.58 (1.13) | 11.99 (5.38) |
| Brain oedema | 6 | 9.13 (4.10,20.33) | 9.11 (4.10,20.27) | 43.31 | 3.19 (0.98) | 9.11 (4.09) |
| Mediastinal haematoma | 6 | 674.60 (298.15,1526.38) | 673.35 (298.03,1521.35) | 3875.18 | 9.34 (1.68) | 647.82 (286.31) |
| Haemodynamic instability | 6 | 15.78 (7.08,35.15) | 15.75 (7.08,35.04) | 82.81 | 3.98 (1.25) | 15.73 (7.06) |
| Catheter site discharge | 6 | 108.85 (48.74,243.09) | 108.65 (48.72,242.29) | 635.93 | 6.75 (1.63) | 107.97 (48.35) |
| Retroperitoneal haematoma | 6 | 50.96 (22.85,113.66) | 50.87 (22.84,113.28) | 292.47 | 5.66 (1.55) | 50.72 (22.74) |
| Procedural hypotension | 6 | 126.12 (56.45,281.78) | 125.89 (56.43,280.84) | 737.96 | 6.97 (1.64) | 124.98 (55.94) |
| Coagulopathy | 5 | 5.62 (2.34,13.51) | 5.61 (2.34,13.47) | 18.94 | 2.49 (0.49) | 5.61 (2.33) |
| Drug specific antibody present | 5 | 14.86 (6.18,35.73) | 14.84 (6.18,35.63) | 64.47 | 3.89 (0.99) | 14.82 (6.16) |
| Mouth haemorrhage | 5 | 12.87 (5.35,30.95) | 12.85 (5.35,30.86) | 54.61 | 3.68 (0.93) | 12.84 (5.34) |
| Ventricular fibrillation | 5 | 8.43 (3.51,20.27) | 8.42 (3.51,20.22) | 32.68 | 3.07 (0.73) | 8.42 (3.50) |
| Coronary artery dissection | 5 | 110.79 (45.95,267.13) | 110.62 (45.94,266.36) | 539.68 | 6.78 (1.34) | 109.92 (45.59) |
| Shock haemorrhagic | 5 | 12.03 (5.00,28.94) | 12.02 (5.00,28.86) | 50.47 | 3.59 (0.90) | 12.01 (4.99) |
| Incision site haemorrhage | 5 | 111.37 (46.19,268.52) | 111.20 (46.18,267.75) | 542.52 | 6.79 (1.34) | 110.49 (45.83) |
| Cerebral haematoma | 5 | 27.81 (11.56,66.92) | 27.77 (11.56,66.73) | 128.84 | 4.79 (1.17) | 27.73 (11.53) |
| Coronary artery restenosis | 5 | 143.81 (59.60,347.02) | 143.59 (59.59,346.02) | 702.08 | 7.15 (1.35) | 142.40 (59.01) |
| Dialysis | 5 | 6.55 (2.72,15.75) | 6.54 (2.72,15.71) | 23.47 | 2.71 (0.59) | 6.54 (2.72) |
| Device occlusion | 5 | 7.11 (2.96,17.09) | 7.10 (2.96,17.04) | 26.18 | 2.83 (0.64) | 7.09 (2.95) |
| Anisocoria | 4 | 49.43 (18.52,131.98) | 49.37 (18.52,131.66) | 189.03 | 5.62 (0.92) | 49.23 (18.44) |
| Creatinine renal clearance decreased | 4 | 18.97 (7.11,50.59) | 18.94 (7.11,50.47) | 67.91 | 4.24 (0.75) | 18.92 (7.09) |
| Haemoperitoneum | 4 | 34.43 (12.90,91.89) | 34.39 (12.90,91.67) | 129.43 | 5.10 (0.87) | 34.32 (12.86) |
| Haemothorax | 4 | 24.39 (9.14,65.07) | 24.36 (9.14,64.91) | 89.48 | 4.60 (0.81) | 24.33 (9.12) |
| Subdural haemorrhage | 4 | 26.92 (10.09,71.82) | 26.88 (10.09,71.65) | 99.54 | 4.75 (0.83) | 26.84 (10.06) |
| Tachypnoea | 4 | 5.70 (2.14,15.20) | 5.69 (2.14,15.16) | 15.47 | 2.51 (0.26) | 5.69 (2.13) |
| Spinal cord haemorrhage | 4 | 191.36 (71.39,512.99) | 191.13 (71.38,511.74) | 748.16 | 7.56 (0.99) | 189.02 (70.51) |
| Puncture site reaction | 4 | 1035.11 (377.06,2841.57) | 1033.84 (377.04,2834.74) | 3891.51 | 9.93 (0.98) | 974.82 (355.10) |
| Pulseless electrical activity | 4 | 15.38 (5.77,41.03) | 15.36 (5.77,40.93) | 53.67 | 3.94 (0.70) | 15.35 (5.76) |
| Intracranial haematoma | 4 | 130.62 (48.81,349.56) | 130.47 (48.81,348.71) | 510.00 | 7.02 (0.98) | 129.48 (48.39) |
| Embolism | 4 | 8.90 (3.34,23.74) | 8.89 (3.34,23.68) | 28.01 | 3.15 (0.49) | 8.89 (3.33) |
| Incorrect drug administration rate | 4 | 19.16 (7.18,51.12) | 19.14 (7.18,50.99) | 68.70 | 4.26 (0.76) | 19.12 (7.17) |
| Infusion site extravasation | 4 | 12.16 (4.56,32.43) | 12.15 (4.56,32.36) | 40.89 | 3.60 (0.62) | 12.14 (4.55) |
| Aortic dissection | 3 | 13.21 (4.26,41.01) | 13.20 (4.26,40.93) | 33.81 | 3.72 (0.26) | 13.19 (4.25) |
| Arteriospasm coronary | 3 | 14.99 (4.83,46.51) | 14.97 (4.83,46.42) | 39.09 | 3.90 (0.29) | 14.96 (4.82) |
| Atrioventricular block complete | 3 | 8.42 (2.71,26.13) | 8.41 (2.71,26.08) | 19.59 | 3.07 (0.12) | 8.41 (2.71) |
| Cerebral ischaemia | 3 | 10.46 (3.37,32.46) | 10.45 (3.37,32.40) | 25.63 | 3.38 (0.19) | 10.45 (3.37) |
| Contrast media reaction | 3 | 24.19 (7.79,75.12) | 24.17 (7.79,74.97) | 66.55 | 4.59 (0.39) | 24.14 (7.78) |
| Coronary artery bypass | 3 | 9.13 (2.94,28.34) | 9.12 (2.94,28.28) | 21.69 | 3.19 (0.15) | 9.12 (2.94) |
| Pancreatitis haemorrhagic | 3 | 115.63 (37.13,360.07) | 115.52 (37.13,359.37) | 338.29 | 6.84 (0.51) | 114.75 (36.85) |
| Prothrombin time prolonged | 3 | 8.76 (2.82,27.18) | 8.75 (2.82,27.13) | 20.59 | 3.13 (0.13) | 8.75 (2.82) |
| Pupil fixed | 3 | 31.31 (10.08,97.23) | 31.28 (10.08,97.04) | 87.78 | 4.96 (0.42) | 31.23 (10.05) |
| Ventricular hypertrophy | 3 | 20.54 (6.62,63.76) | 20.52 (6.62,63.63) | 55.64 | 4.36 (0.36) | 20.50 (6.60) |
| Stent placement | 3 | 7.01 (2.26,21.75) | 7.00 (2.26,21.71) | 15.44 | 2.81 (0.04) | 7.00 (2.26) |
| Coronary artery reocclusion | 3 | 400.17 (127.31,1257.91) | 399.80 (127.32,1255.45) | 1166.09 | 8.61 (0.52) | 390.67 (124.28) |
| Bloody discharge | 3 | 50.51 (16.26,156.97) | 50.47 (16.26,156.66) | 145.04 | 5.65 (0.47) | 50.32 (16.19) |
| Troponin increased | 3 | 8.11 (2.61,25.16) | 8.10 (2.61,25.12) | 18.67 | 3.02 (0.10) | 8.10 (2.61) |
| Anti-platelet antibody positive | 3 | 306.72 (97.87,961.20) | 306.44 (97.89,959.31) | 897.21 | 8.23 (0.53) | 301.05 (96.06) |
| Iatrogenic injury | 3 | 41.37 (13.32,128.53) | 41.34 (13.32,128.28) | 117.80 | 5.37 (0.45) | 41.24 (13.28) |
| Ischaemia | 3 | 11.82 (3.81,36.68) | 11.81 (3.81,36.61) | 29.66 | 3.56 (0.23) | 11.80 (3.80) |
| Heparin-induced thrombocytopenia | 3 | 11.26 (3.63,34.96) | 11.25 (3.63,34.89) | 28.01 | 3.49 (0.21) | 11.25 (3.62) |
| Tracheal haemorrhage | 3 | 162.09 (51.97,505.56) | 161.95 (51.98,504.57) | 475.35 | 7.33 (0.52) | 160.43 (51.44) |
| Product container issue | 3 | 7.24 (2.33,22.47) | 7.24 (2.33,22.43) | 16.12 | 2.85 (0.06) | 7.23 (2.33) |
| Myocardial necrosis marker increased | 3 | 24.95 (8.04,77.46) | 24.93 (8.04,77.31) | 68.80 | 4.64 (0.39) | 24.89 (8.02) |

Abbreviation: PT, preferred term; ROR, reporting odds ratio; CI, confidence interval; PRR, proportional reporting ratio; χ^2^, Chi-Square; IC, information component; IC025, the lower limit of the 95% CI of the IC; EBGM, empirical Bayesian geometric mean; EBGM05, the lower limit of the 95% CI of EBGM. Signals are detected when all the following criteria are met: N≥3, PRR≥2 and Chi-Square (χ^2^) ≥4, lower limit of 95% CI of ROR>1, IC025>0, EBGM05>2.
